# Structures of the *Staphylococcus aureus* ribosome inhibited by fusidic acid and fusidic acid cyclopentane

**DOI:** 10.1038/s41598-024-64868-x

**Published:** 2024-06-20

**Authors:** Adrián González-López, Daniel S. D. Larsson, Ravi Kiran Koripella, Brett N. Cain, Martin Garcia Chavez, Paul J. Hergenrother, Suparna Sanyal, Maria Selmer

**Affiliations:** 1 Department of Cell and Molecular Biology, Uppsala University, BMC, P.O. Box 596, 75124 Uppsala, Sweden; 2 Department of Chemistry, University of Illinois at Urbana−Champaign, Urbana, IL 61801 USA; 3 Present Address: Robert P. Apkarian Integrated Electron Microscopy Core, Emory University, Atlanta, USA

**Keywords:** Fusidic acid, Ribosome, EF-G, Cryo-EM, Elongation factor G, Drug discovery, Structural biology, Electron microscopy

## Abstract

The antibiotic fusidic acid (FA) is used to treat *Staphylococcus aureus* infections. It inhibits protein synthesis by binding to elongation factor G (EF-G) and preventing its release from the ribosome after translocation. While FA, due to permeability issues, is only effective against gram-positive bacteria, the available structures of FA-inhibited complexes are from gram-negative model organisms. To fill this knowledge gap, we solved cryo-EM structures of the *S. aureus* ribosome in complex with mRNA, tRNA, EF-G and FA to 2.5 Å resolution and the corresponding complex structures with the recently developed FA derivative FA-cyclopentane (FA-CP) to 2.0 Å resolution. With both FA variants, the majority of the ribosomal particles are observed in chimeric state and only a minor population in post-translocational state. As expected, FA binds in a pocket between domains I, II and III of EF-G and the sarcin-ricin loop of 23S rRNA. FA-CP binds in an identical position, but its cyclopentane moiety provides additional contacts to EF-G and 23S rRNA, suggesting that its improved resistance profile towards mutations in EF-G is due to higher-affinity binding. These high-resolution structures reveal new details about the *S. aureus* ribosome, including confirmation of many rRNA modifications, and provide an optimal starting point for future structure-based drug discovery on an important clinical drug target.

## Introduction

Fusidic acid (FA) is a bacteriostatic steroid antibiotic that inhibits bacterial protein synthesis. Due to low uptake^1,2^ and efflux through the outer membrane of gram-negative bacteria^3^, FA is effective mainly against gram-positive bacteria and used routinely against topical *Staphylococcus aureus* infections since the 1960s^4^. However, due to resistance development^5,6^, FA is usually recommended at high doses and in combination with other antibiotics ^7^. The development of new FA derivatives has been largely unsuccessful, as modifications of the drug usually come at the cost of potency^8,9^.

FA targets elongation factor G (EF-G), the GTPase factor that catalyzes translocation of mRNA and tRNAs on the ribosome^10^. FA inhibits the release of EF-G from the ribosome and thereby stalls translation^11,12^. Translocation requires two major conformational changes of the ribosome: intersubunit rotation and rotation of the head of the small ribosomal subunit (SSU), known as swiveling^13,14^. FA also locks EF-G in the recycling step of translation^15,16^, where EF-G together with ribosome recycling factor separates the ribosomal subunits after termination^17^. The binding site of FA is a pocket between domains I-III of EF-G and the sarcin-ricin loop (SRL) of 23S rRNA, which is formed after GTP hydrolysis. FA binding seems to stabilize an intermediate between the GTP and GDP conformation of EF-G, where switch I is in GDP conformation but switch II remains in GTP conformation, which disables the necessary conformational changes for EF-G release^18^.

High-resolution structural information for the FA-inhibited ribosome was first obtained from X-ray crystallography with EF-G and ribosomes from *Thermus thermophilus*^18–22^, and more recently from cryo-EM studies with components from *Escherichia coli*^23^. From the actual FA target pathogen, *S. aureus*, there are only structures of EF-G without the ribosome^24^. EF-G from *S. aureus* shows 60% sequence identity to EF-G from *E. coli* and 62% sequence identity to EF-G from *T. thermophilus*, and 23S rRNA from these species share about 70% sequence identity. Thus, understanding of the resulting structural differences can have an impact on future drug discovery projects.

The FA-locked structures have exhibited two different ribosomal states. One is the post-translocational state (POST)^21^, where the ribosome has completed translocation, and tRNAs are bound in the canonical P and E sites. The other is an intermediate so-called chimeric state (CHI) of translocation, in which the head of the small ribosomal subunit (SSU) remains swiveled, the tRNAs make A- and P-site interactions with the mRNA codon and the SSU head, and P- and E-site interactions with the 30S body and the 50S^14,20^. In both states, EF-G is in close contact with 23S rRNA, 16S rRNA, uL6, uS12, mRNA, and P-site tRNA.

There are 3 main types of FA resistance observed in *Staphylococcus sp*. The *fusA* mutants have mutations in the drug target, EF-G^5,25^. FusB-type resistance is caused by a *fusB*-encoded resistance protein (with homologous variants FusC, FusD, and FusF) that binds to EF-G and causes resistance without direct interaction with the antibiotic^26,27^. The *fusE* variants carry truncations or loss-of-function mutations in the *rplF* gene, encoding ribosomal protein uL6, which interacts with EF-G^6^.

Here, we set out to determine high-resolution cryo-EM structures of the clinical FA drug target, the *S. aureus* 70S ribosome where EF-G is locked with FA or a recently developed FA derivative. These structures allowed comparison with ribosome-EF-G-FA structures from other bacteria, a revised analysis of known *fusA* resistance mutations and refinement of a high-quality model that can be used for the design of new FA derivatives or analogs, as well as other drugs targeting the *S. aureus* ribosome.

## Results and discussion

### Structure of the *S. aureus* ribosome

**Figure 1:**
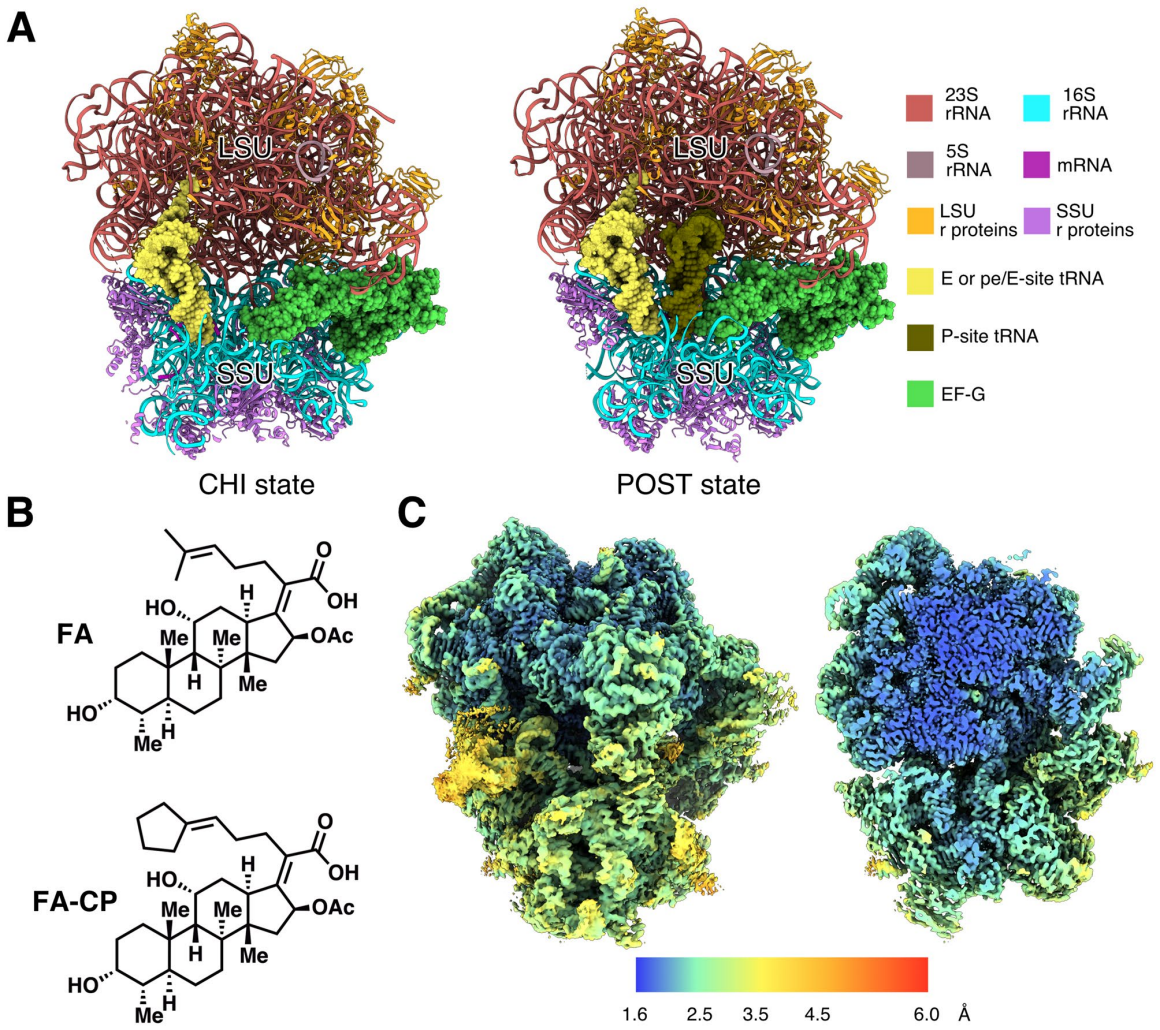
Structures of *S. aureus* ribosomes in complex with mRNA, tRNA, EF-G, and FA/FA-CP. (**A**) Composition of the different complexes. Large subunit (LSU) and small subunit (SSU) are indicated. (**B**) Chemical structure of FA and FA-CP. (**C**) Local resolution map over the surface and a central slice on the highest resolution map (2.0 Å FA-CP CHI state).

We assembled a minimal high-occupancy FA-locked *S. aureus* ribosome complex for structure determination. *S. aureus* 70S ribosomes were mixed with a short synthetic mRNA, *E. coli* tRNA^fMet^, and *S. aureus* EF-G in presence of FA and GTP and imaged by cryogenic electron microscopy (cryo-EM). Image processing using cryoSPARC^28^ produced two different ribosome reconstructions in complex with EF-G bound to FA: A post-translocational state (POST) at 3.1 Å resolution, and a chimeric pe/E state (CHI) at 2.5 Å (Fig. 1A).

We then applied the same methodology to the recently developed FA derivative fusidic acid cyclopentane (FA-CP, Fig. 1B)^29^, which has equivalent potency against *S. aureus* as FA, but a better resistance profile. This resulted in a 2.0-Å resolution CHI state reconstruction and a 2.4-Å POST state.

The reconstructed maps are very similar, the main differences between the CHI and POST states are the head swivel of the SSU, and the resulting changes in interactions with the tRNAs and EF-G. In all complexes, the ribosomal A-site is occupied by domain IV of EF-G. The POST state contains two tRNAs in the P- and E sites, whereas the CHI state only has one tRNA bound in pe/E state (Fig. 1A). The pe/E tRNA makes P-site interactions with the codon on the mRNA and the SSU head (16S nucleotides 1349–1352 and 1238–1241), but E-site interactions with the body of 16S (nucleotides 700–704), and with the 50S subunit. The mRNA Shine-Dalgarno sequence is base paired with the anti-Shine-Dalgarno 1546C-1551U of 16S rRNA in both CHI structures. In the POST structure, however, both the mRNA and the 16S rRNA are disordered in this region. The CHI state is the most abundant in our sample, and only a minor population is in POST state. In the FA data, 60% of the ribosomes are bound to EF-G, and 93% of those are in CHI state. In the FA-CP data, 72% of the 70S particles contain EF-G, out of which 88% are in CHI state. The occupancy of FA and FA-CP is assumed to be the same as for EF-G, since EF-G would dissociate from the ribosome in absence of inhibitor.

The map quality of FA-CP CHI complex was excellent with a majority of the large subunit reaching 1.6 Å (Fig. 1C). The high resolution allowed the refinement of a high-quality model of the *S. aureus* 70S ribosome (Table 1), identification of many rRNA modifications, and correction of modeling errors in previous lower-resolution structures (*e.g.* a D159-A160 cis-peptide bond in uL3, Figure S1). This model was subsequently refined into the FA-CP POST and FA-CHI maps. Due to map anisotropy and lower quality, no modelling of the FA POST state was done.

**Table 1:** Cryo-EM refinement parameters and model validation.

	FA CHI	FA-CP CHI	FA-CP POST
Map refinement			
Number of particles	178 679	303 005	40 800
Map resolution, half-map FSC0.143 [Å]	2.49	2.02	2.44
Model statistics			
*Model composition*			
Total non-hydrogen atoms	146 593	149 701	149 413
RNA residues	4585	4585	4645
Protein residues	6091	6091	6103
Waters	0	3169	1619
Mg ions	169	169	145
*General*			
Map-model FSC0.5 [Å]	2.5	2	2.4
Map-model CCmask	0.89	0.88	0.9
MolProbity score	1.25	1.26	1.26
Clash score	2.89	3.46	2.82
RMSD bond length [Å]	0.005	0.004	0.006
RMSD bond angles [°]	0.633	0.632	0.682
Mean B-factor (protein/nucleotide/ligand/water)	70.7/74.1/51.2/-	40.9/35.1/19.7/8.4	56.9/61.8/40.8/38.9
*Protein geometry*			
Rotamer outliers [%]	1.05	0.99	1.24
Cß deviations > 0.25 Å [%]	0	0	0
Ramachandran [%] (Fav./Allow./Outl.)	97.1/2.9/0.02	97.3/2.7/0.02	97.4/2.6/0.03
Rama-Z (whole/helix/sheet/loop)	0.05/0.88/-0.20/-0.38	0.78/1.80/-0.12/-0.04	0.76/1.74/-0.17/0.00
CaBLAM [%] (Outl./Disf.)	0.98/5.14	0.92/5.31	1.00/5.07
CA Geometry outliers [%]	0.46	0.49	0.51
EMRinger score	4.21	5.48	4.32
*Nucleic acid geometry*			
Backbone bond length outliers [%]	0	0	0
Backbone bond angle outliers [%]	0	0	0
Sugar puckers outliers [%]	0.48	0.50	0.43
Backbone bond torsion suite outliers [%]	11.9	11.9	12.8

### *S. aureus* EF-G on the ribosome

The binding and overall conformation of the FA-bound *S. aureus* EF-G on the ribosome agrees with previous cryo-EM and X-ray crystallography structures of FA-locked complexes from gram-negative bacteria^18–21,23^*.* Overall, our POST structure is most similar to the *T. thermophilus* structure in the same state (PDBID: 4WQF, TtPOST)^19^, whereas the CHI structure is more similar to CHI state structures that have both ap/P and pe/E tRNAs, from *E. coli* (PDBID: 7N2C, EcCHI)^23^ and *T. thermophilus* (PDBID: 4W29, TtCHI)^20^. Other CHI-state structures that only have one tRNA in the pe/E site (*e.g.* PDB ID: 4V9L)^21^ show a different degree of head swivel.

The conformation of EF-G is very similar in the CHI and POST states (RMSD: 0.382 Å over 666 Cα atoms of EF-G). The largest difference is found in a loop at the tip of domain IV (residues 498–505), due to additional interactions with the mRNA and P-site tRNA in the POST structure.

**Figure 2:**
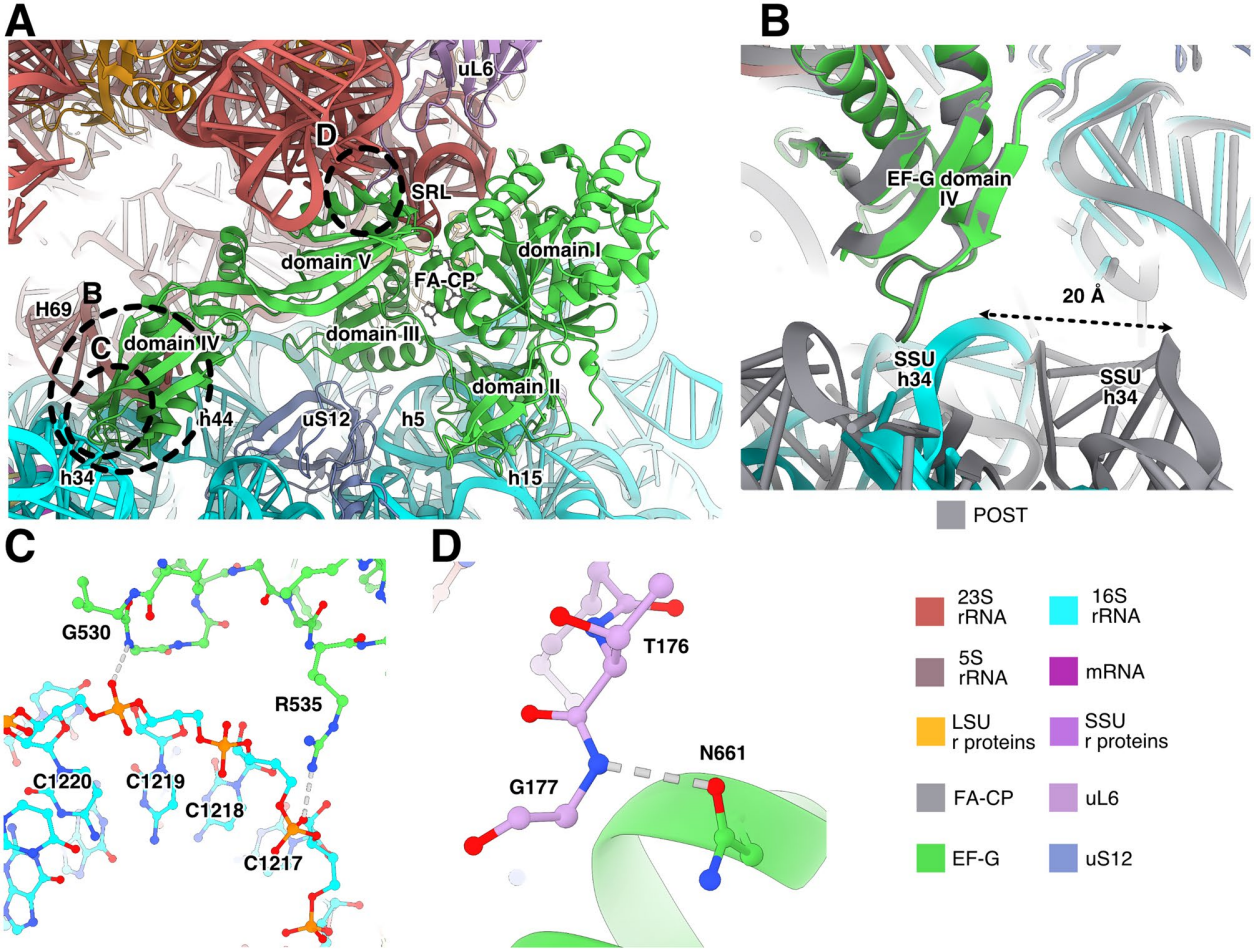
Interactions of EF-G with the *S. aureus* ribosome. (**A**) Binding site of EF-G in the ribosomal CHI state. Interacting helices from 23S rRNA are indicated with “H” and helices from 16S rRNA with “h”. (**B)** Differences between the SSU contacts of EF-G in POST (gray) and CHI state. The SSU head swivel brings 16S rRNA helix 34 in contact with EF-G. (**C**) Interactions of EF-G with helix 34 in the CHI state. (**D**) Interaction between EF-G and uL6 shown for the CHI state.

EF-G interacts with both 23S and 16S rRNA, as well as with ribosomal proteins uL6 and uS12 (Fig. 2A). The main contacts with 23S rRNA are between domains I and V of EF-G (residues H18, H85, S660 and Q663) and the SRL proximal to the FA binding site (Fig. 2A). Domain IV of EF-G interacts with 23S and 16S rRNA at the intersubunit interface, where R498 contacts helix 69 of 23S rRNA and helix 44 of 16S rRNA (Figure S2). In the *E. coli* structure, the equivalent residue (K507) does not reach the corresponding interface. In the CHI structures, we observe an additional interaction, not observed in previous structures, between S586 in EF-G and C1941 (C1914 in *E. coli*), in the hairpin loop of helix 69 of 23S rRNA at intersubunit bridge B2a (Figure S2). In the CHI state, C1941 is observed in two conformations, it either interacts with EF-G (Figure S2A) or stacks with helix 69 (Figure S2B). The previous nucleotide, A1940 (A1913 in *E. coli*), can also adopt different conformations depending on the state of the ribosome. In our structures, it stacks with A1503 (A1492 in *E. coli*) of 16S rRNA when EF-G contacts its backbone. In absence of EF-G, it instead forms a base interaction with A-site tRNA (*e.g.* in PDB 7K00^30^).

Domain II of EF-G binds to 16S rRNA helix 5 (residue K324) and helix 15 (residues Y340, R349, R351, R354). In EcCHI, the contact through R349 is not present, since the corresponding sidechain (R357 in *E. coli*) points in the opposite direction and is far from the RNA. The equivalent of residue 340 is a threonine in EcCHI (T348) that does not reach the RNA (Figure S3A-B). In the CHI structure, EF-G makes additional contacts with helix 34 in the head region of 16S rRNA (Fig. 2B,C). This results in a 10% larger hidden surface area of EF-G compared to the POST state, suggesting that higher stability causes its higher abundance. In fact, in the recent time-resolved cryo-EM studies of EF-G translocation in *E. coli*^31,32^, EF-G was only present in pre-translocation and chimeric states. The ribosomal POST conformation was only observed after EF-G dissociation, suggesting that EF-G naturally leaves the ribosome before the POST conformation is reached. Furthermore, in cryo-EM structures of FA-inhibited *E. coli* ribosomes, only the CHI state was observed^23^. In our data, however, we do observe POST state, indicating that FA inhibition allows time for the SSU head to back-swivel in presence of EF-G.

Differently from TtPOST and EcCHI, EF-G contacts uL6 through a polar interaction between N661 and the backbone of T176 (Fig. 2D, Figure S3C, D). This can explain why truncations of the C-terminus of uL6 (*fusE* mutations) provide low-level resistance to FA, since this will weaken the interactions of EF-G with the ribosome and facilitate its release in presence of FA.

EF-G also interacts with uS12 through residues D421, T441 and E443 in domain III (Fig. 2A). In TtPOST, the interactions are similar, but residue 443 is a proline that cannot form the equivalent interaction. The interaction with uS12 in EcCHI is slightly different, *e.g.* the interaction of T441 is replaced by the equivalent residue of *S. aureus* G446 (N454) (Figure S3E, F).

### Binding site of FA and FA-CP

**Figure 3:**
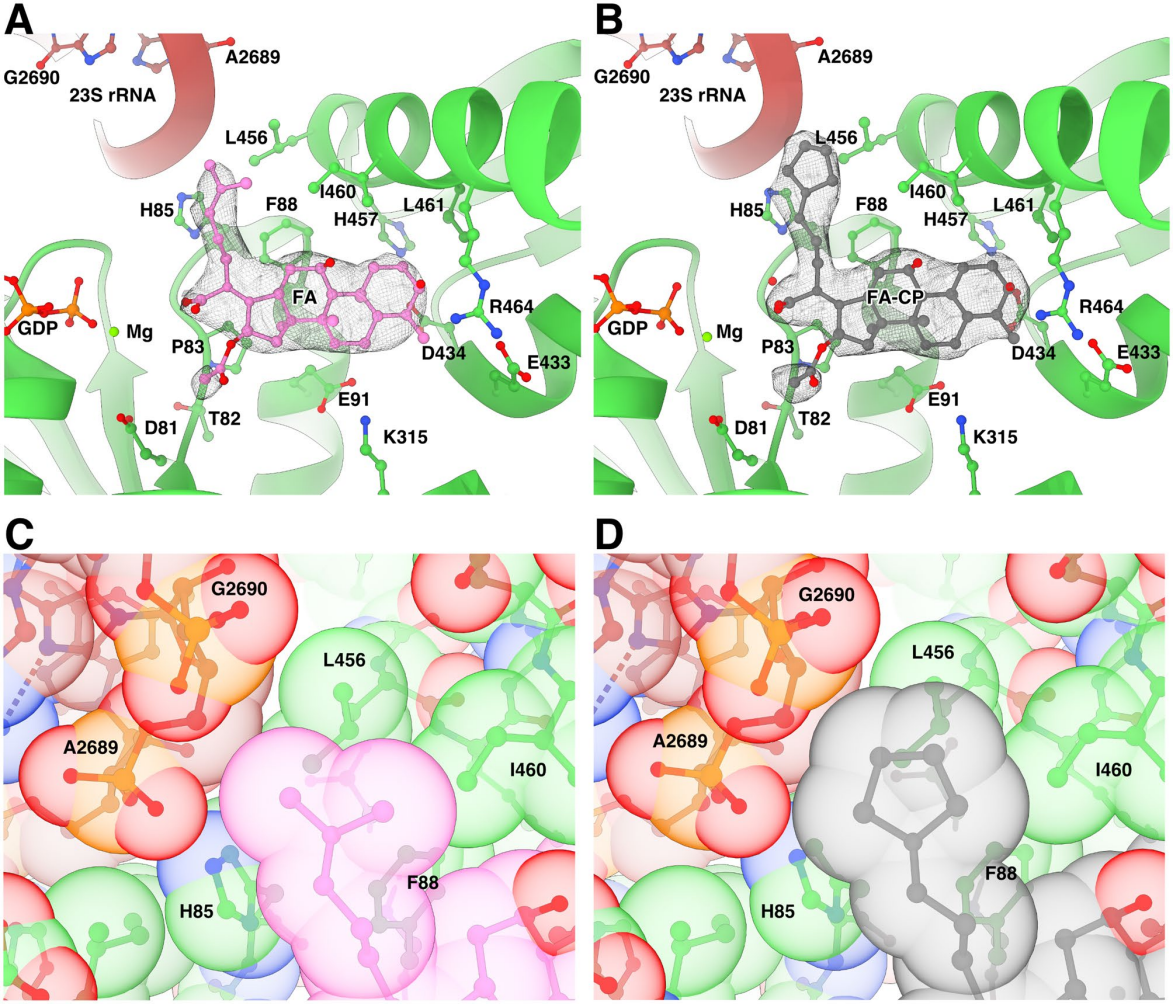
Binding site of FA and FA-CP. (**A**–**B**) Segmented local filtered maps around FA (**A**) or FA-CP (**B**) in CHI state. Side chains within 4.5 Å of FA or FA-CP are shown as sticks. (**C**–**D**) Close-up of the packing of the dimethyl alkene side chain of FA (**C**) and the cyclopentane variant in FA-CP (**D**) within the binding pocket, showed with transparent ball representation at van der Waals radii.

FA and FA-CP bind next to GDP and magnesium in a pocket between domains I-III of EF-G and the SRL of 23S (Fig. 3A, B, Figure S4A), exposed to the solvent at the ribosomal subunit interface. The estimated local resolution of this region in the FA-CP map is around 2.5 Å, Figure S4B). The main interactions of EF-G with FA are formed by switch II (residues 80–90) in domain I and helix 9 (residues 455–470) in domain III. Before GTP hydrolysis, switch I (residues 39–63) occupies this site to interact with the gamma phosphate of GTP, impeding FA binding. After GTP hydrolysis, switch I becomes disordered and switch II changes conformation. FA locks switch II in the GTP conformation, stabilizing EF-G’s interactions with the ribosome (*e.g.* H85 with 23S) and inhibiting its release (Figure S5).

Both FA and FA-CP have a similar density level as the surrounding protein, as well as similar B-factors, in agreement with a full occupancy of the antibiotics. In addition, our maps clearly show the extra density for the carbons of the cyclopentane moiety of FA-CP (Fig. 3A, B, Figure S4A), forming tighter interactions with H85, L456 and the SRL (Fig. 3C, D, Figure S4C-D). The cyclopentane moiety extends 0.3 Å closer to both the SRL backbone and H85 (Figure S4C, D). The interactions formed by the additional atoms in FA-CP presumably lead to increased affinity and further stabilization of the FA-bound conformation of EF-G. In line with previous structure–activity relationships, this region of the binding pocket appears to be most amenable to structure modifications as other relatively lipophilic moieties such as cyclohexane and tetrahydropyran rings can be placed in the same location with no detriment to antimicrobial activity in *S. aureus*^29^. Future projects aiming for improved activity against *S. aureus* may be successful in identifying higher affinity derivatives by thoroughly probing this area to further explore interactions with EF-G and rRNA.

**Figure 4:**
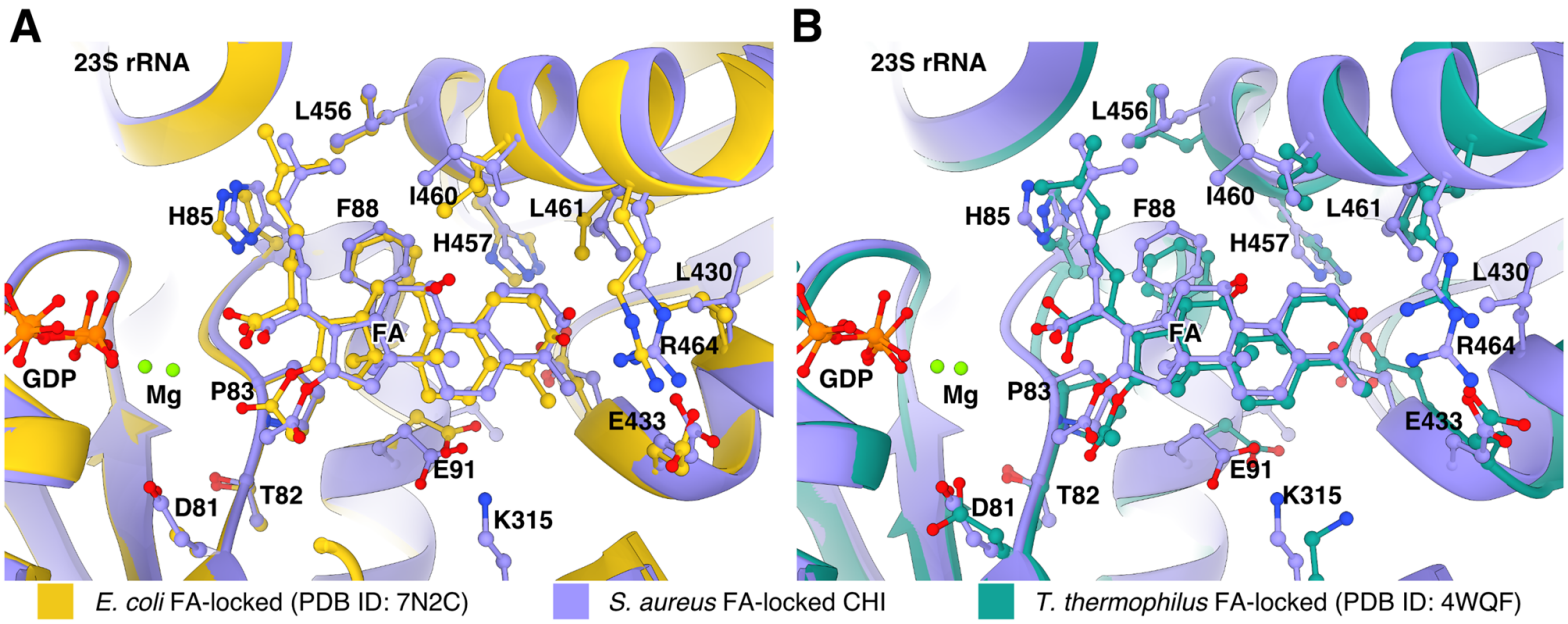
Comparison of the FA binding site in *S. aureus* complexes with previously available structures. Structures were aligned based on EF-G and side chains within 4.5 Å of FA are shown as sticks. (**A**) Overlay of the FA binding site in the *S. aureus* CHI state and EcCHI (PDB ID: 7N2C). (**B**) Overlay of the FA binding site in the *S. aureus* CHI state and TtPOST (PDB ID: 4WQF).

In comparison with previously published structures^19,23^, FA binds in the same way. There are minor differences in the modeled sidechain conformations in the EcCHI structure, particularly for I460 (Fig. 4A). On the other hand, in TtPOST, residues L456 and I460 have different rotamers, switch II has a slightly different orientation with H85 farther from interaction with 23S, and helix 9 from domain III is tilted, resulting in a 2 Å difference in the Cα position of R464 (Fig. 4B).

### *fusA* mutants

**Figure 5:**
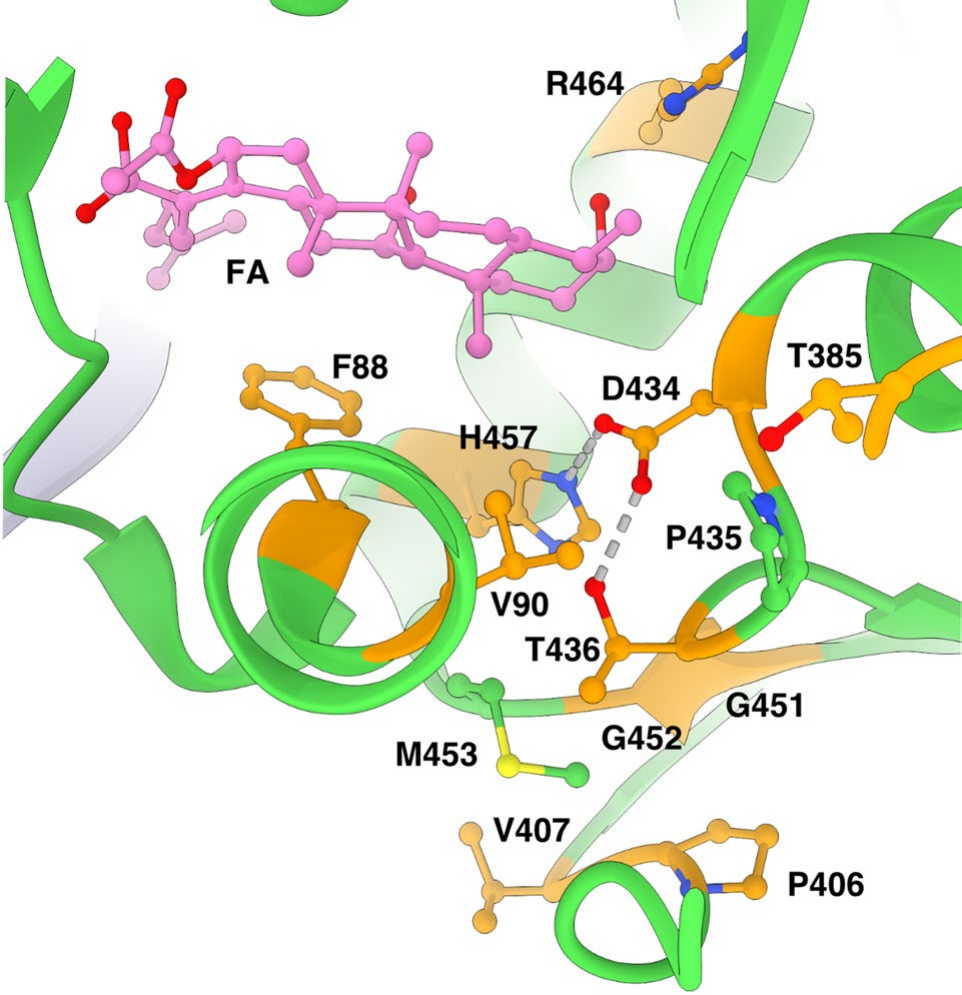
Network of interactions in EF-G around the FA binding site. Sites of *fusA* resistance mutations are shown in orange.

Structures of FA-locked ribosome complexes have demonstrated that FA-inhibition requires drug binding to a certain EF-G conformation with specific interactions with the ribosome. FA resistance mutations in EF-G (*fusA*-type) from clinical isolates and lab studies^6,33–35^ have been found to affect all of these aspects, and have thus been classified into four groups according to their expected mechanism of rescue: effects on FA binding, ribosome-EF-G interactions, EF-G conformation or EF-G stability^24^. Based on our high-resolution structures, we analyze seven different individual sites of mutations where FA-CP gives lower minimum inhibitory concentrations than FA^29^. Around the FA binding site, the strong F88L mutation and the milder R464A would weaken direct EF-G-FA interactions (Fig. 5). The higher affinity of FA-CP may partially compensate for the absence of these interactions. The potent resistance mutations D434N and H457L/N/Y and the milder T436I would break the hydrogen-bond network between D434, T436 and H457 (at 4.3 Å distance of F88 in domain I), which bring together two helices at the drug-facing surface of domain III. Additional mutations in domain III are connected to this network by hydrophobic interaction directly: V90I; via M453: P406L (tested with FA-CP), V407F, G451V and G452C/SV; or via P435: T385N in domain II (tested with FA-CP) (Fig. 5). We thus confirm that interactions in the core of domain III are important for FA potency, directly and through interactions with domains I and III. We also conclude that tighter binding of FA-CP seems to lead to increased stability of the FA-locked conformation; allowing FA-CP to partially compensate for mutation-induced non-optimal interactions in this core. It remains to be tested whether FA-CP also shows increased potency against the milder resistance mutations in domain V of EF-G.

### *S. aureus* ribosomal RNA modifications

**Figure 6:**
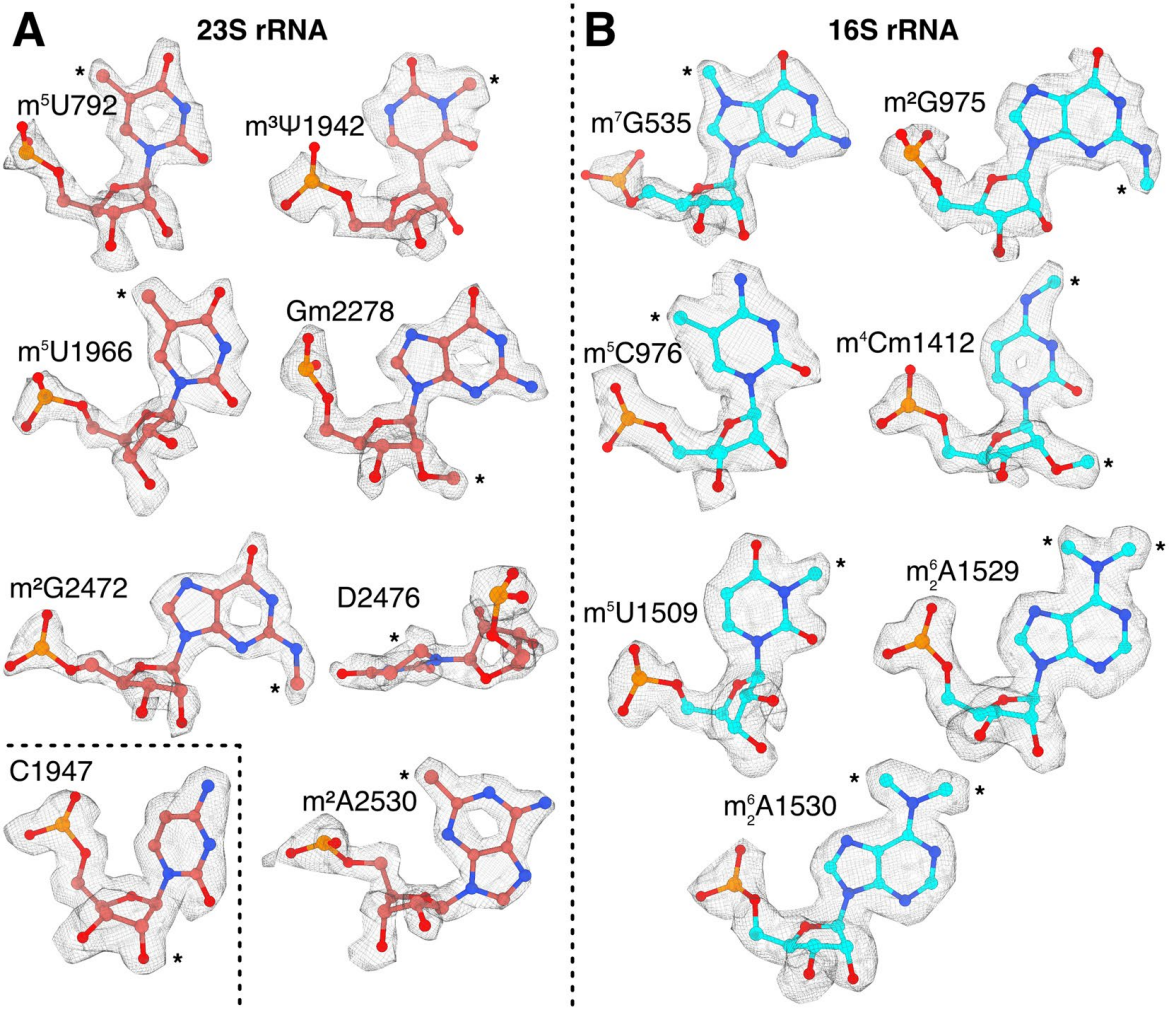
Identification of post-transcriptionally modified rRNA nucleotides in local filtered maps of the FA-CP CHI complex of the *S. aureus* ribosome. Asterisks indicate sites of modification. (**A**) Identified rRNA modifications in 23S rRNA. The inset shows that C1947 lacks 2′O methylation. (**B**) rRNA modifications in 16S rRNA.

Posttranscriptional modifications of rRNA are present in all kingdoms of life, but the details of modifications vary, even between closely related bacteria. Due to the lack of mass spectrometry data on rRNA modifications in *S. aureus*, the previous assignment was based on a 3.2 Å resolution structure^36^. We here used our higher resolution structural data to gain further insights on the native rRNA modifications in *S. aureus*. Based on the analysis of the 2.0 Å cryo-EM map and the comparison with *E. coli* rRNA modifications, 14 rRNA modifications could be identified (Fig. 6) including two novel modifications of 23S rRNA and three novel modifications of 16S rRNA that previously had not been observed in *S. aureus*. The previously reported 2’-O-methylcytidine in position C1947 of 23S rRNA^36^ is not present in our structure.

For 23S rRNA, we identified a 5-methyluridine in position U792 (helix 35, *E. coli* U747), where the methyl moiety packs against the ribose of C2639. The corresponding modification is made by RlmC in *E. coli*. A BLAST^37^ search against *S. aureus* proteins only gave hits classified as homologs of RlmD, another uridine methylase that modifies U1939. We also observed C1966 (equivalent position in *S. aureus*) to be methylated. In other gram-positive bacteria, such as *Bacillus subtilis*, both of these modifications are performed by the dual-specificity enzyme RlmCD^38^. Our observations suggest that this also occurs in *S. aureus*, as earlier proposed without observation of the m^5^U792 modification^36^.

Additionally, we found clear density for a non-planar base at position 2476, located at the peptidyl-transfer center, which suggests the presence of 5,6-dihydrouridine (*E. coli* D2449).

We also found density for a methylation of N3 of at position 1942, located in helix 69 of 23S. In *E. coli*, this is an N3-methylpseudouridine. There are homologues of RluD and RlmH in *S. aureus*, corroborating that the same modifications are present in *S. aureus*.

For 16S rRNA, we found a N2-methylguanosine at position G975 and 5-methylcytidine at C976, located at the loop of helix 31 (same modification in *E. coli* G966 and C967), part of the P-site. The methyl group in G975 stacks against K131 of uS9, which is interacting directly with the P-site tRNA next to the anticodon loop.

Lastly, we also found a 5-methyluridine at position U1509, in helix 44 (same modification in *E. coli* 1498). This modification is also located on the P-site, next to the mRNA codon-anticodon interaction, where also C1412 is modified to N4,O2′-dimethylcytidine.

A summary of all the rRNA modifications that were confidently identified from the density can be found in Table 2.

**Table 2:** *S. aureus* rRNA modifications identified in the cryo-EM map.

Position *S. aureus*	Position *E. coli*	Modification	*E. coli* enzyme	Probable *S. aureus* enzyme (UniProt ID)
23S				
792	747	5-Methyluridine	RlmC	RlmCD (A0A0U1MPM5)
1942	1915	N5-Methylpseudouridine	RluD and RlmH	RluD (Q2FX95) and RlmH (Q2G252)
1966	1939	5-Methyluridine	RlmD	RlmCD (A0A0U1MPM5)
2278	2251	2′O-Methylguanosine	RlmB	RlmB (Q2G2M3)
2472	2445	N2-Methylguanosine	RlmKL	RlmK (A0A0U1MLF0)
2476	2449	5,6-Dihydrouridine	Unknown	–
2530	2503	2′-Methyladenosine	RlmN	RlmN (Q2FZ66)
16S				
535	527	N7-Methylguanosine	RsmG	RsmG (Q2FUQ4)
975	966	N2-Methylguanosine	RsmD	RsmD (W8TQE6)
976	967	5-Methylcytidine	RsmB	RsmB (Q2FZ67)
1412	1402	N4, O 2′-Dimethylcytidine	RsmI and RsmH	RsmI (Q2G1S1) and RsmH (P60393)
1509	1498	5-Methyluridine	RsmE	RsmE (Q2FXZ5)
1529	1518	N6-Dimethyladenosine	RsmA	RsmA (Q2G0T0)
1530	1519	N6-Dimethyladenosine	RsmA	RsmA (Q2G0T0)

## Conclusions

In this work, we have determined high-resolution cryo-EM structures of FA and its derivative FA-CP in complex with the clinical drug target, the *S. aureus* ribosome-EF-G complex. The structures show two different states, a predominant CHI state and a less abundant POST state. In line with other recent cryo-EM studies, our structures suggest that the POST state of EF-G may only be stable in presence of FA, whereas EF-G, during unperturbed translocation, would quickly dissociate from the ribosome.

The first structures of FA-inhibited ribosome complexes were at resolutions where modelling of FA and its interactions with EF-G was challenging^18,20,21^. Using the advances in cryo-EM, our high-resolution reconstructions allow accurate modeling of the FA molecule and its binding site in the clinical target. Moreover, we show that FA-CP binds in an identical position as FA and forms the same interactions with EF-G. In addition, the cyclopentane moiety of FA-CP has closer contacts with two side chains of EF-G and the SRL of 23S rRNA. There is additional space in this part of the binding pocket, which could be explored in future FA derivatives. The observed interactions suggest that the improved resistance profile is due to a higher binding affinity.

The structure also demonstrates how the higher-affinity derivative partially compensates for resistance mutations that weaken the direct FA-EF-G interactions or destabilize domain III and its interactions with domains I and II. Domain III of EF-G appears to have evolved to allow dynamics during the elongation cycle^39^, but its stability is also key to FA inhibition.

The high-resolution maps allowed building and refinement of high-quality models including many of the native rRNA modifications. The structure provides an accurate starting point for structure-guided drug discovery, not only of FA derivatives and analogues, but also of other antibiotics that target the *S. aureus* ribosome.

## Materials and methods

### Cloning, overexpression, and purification of *Staphylococcus* aureus EF-G

*Staphylococcus aureus* EF-G *fusA* gene was amplified using Phusion master mix (Thermo Fisher Scientific, Waltham, MA, USA) from a previous EF-G construct in pET30^24^ with the primers (5′)ATG GCT AGA GAA TTC TCA TTA GAA AAA ACT C and (5′)GTT ATT CAC CTT TAT TTT TCT TGA TAA TAT CTT CAG C and cloned into pEXP5-NT following the manufacturer’s instructions (pEXP5-NT_SaEF-G). The sequence was confirmed by Sanger sequencing. This construct encodes a 6xHis tag with a TEV cleavage site and a linker to the EF-G sequence. After TEV cleavage, only Ser-Leu remains at the N-terminus.

pEXP5-NT_SaEF-G was transformed into *E. coli* BL21 (DE3). An overnight culture was inoculated 1:100 into a 2.8 l baffled flask with 800 ml of LB with 100 µg/ml of ampicillin. Protein expression was induced at an OD_600 nm_ of 0.5–0.6 with 1 mM isopropyl-β-thiogalactopyranoside and the culture was incubated for 16–18 h at 16 °C. The cells were harvested at 7 460 × g for 30 min in a JLA 9.1000 rotor (Beckman Coulter, Brea, California, USA), washed with 150 mM NaCl, and stored at − 20 °C.

The cells were resuspended in lysis buffer (50 mM Tris–HCl pH 7.5, 300 mM NaCl, 5 mM β-mercaptoethanol) with 0.1 % (v/v) Triton X-100, one EDTA-free mini-Complete protease inhibitor tablet (Roche, Basel, Switzerland) and DNase I. Then, they were lysed in a flow cell disruptor (Constant Systems Ltd., Daventry, United Kingdom). The lysate was centrifuged at 26 900 × g for 45 min in an SS-34 rotor (Thermo Fisher Scientific).

The supernatant was filtered by 0.45 µm with a polyethersulfone syringe filter (Sarstedt AG & Co, Nûmbrecht, Germany) and incubated with 2 ml of Ni Sepharose Fast Flow (Cytiva, Uppsala, Sweden) equilibrated with lysis buffer. The column was washed with 25 column volumes of 10 mM imidazole in lysis buffer and the protein was eluted with 500 mM imidazole in lysis buffer. The eluate was exchanged back to lysis buffer using a PD-10 column (Cytiva, Uppsala, Sweden). The 6x-histidine-tag was removed by cleavage with 1:25 molar ratio of TEV protease at 8 °C for 16–18 h followed by reverse IMAC on 1 ml of Ni Sepharose Fast Flow. *S. aureus* EF-G was purified further by gel filtration on a Hiload 16/60 Superdex-200 column (GE Healthcare, Uppsala, Sweden) equilibrated with lysis buffer. The peak fractions were concentrated to 5–10 mg/ml with a 30-KDa cutoff Vivaspin Turbo 15 (Sartorius AG, Göttingen, Germany), flash-frozen in liquid nitrogen, and stored at − 80 °C.

### Ribosome purification

200 ml of *S. aureus* NCTC 8325–4 culture was grown at 37 °C with shaking (200 rpm) in Mueller Hinton Broth (MHB) and harvested in early logarithmic phase (A600 = 1.0 AU/ml). The cells were pelleted by centrifugation at 5000 × g, and weighed approximately 0.5 g. The cell pellet was resuspended in 50 ml of 20 mM Tris–HCl pH 7.5, 100 mM NH_4_Cl, 10 mM magnesium acetate, 0.5 mM EDTA, 3 mM β-mercaptoethanol (BME) and 0.3 μg/ml DNase I supplemented with 600 μl of protease inhibitor cocktail (Roche, Basel, Switzerland) and 5 mg lysostaphin (Sigma-Aldrich, Merck, Darmstadt, Germany) and incubated for 1 h at 37 °C. The cells were then lysed by French press and the cell debris was pelleted and discarded by two centrifugations of 30 min at 48 000 × g using a SS-34 rotor at 4 °C. The supernatant was applied with volume ratio 1:1 on a 1.1 M sucrose cushion in 20 mM Tris–HCl pH 7.5, 0.5 M NH_4_Cl, 10 mM magnesium acetate, 0.5 mM EDTA, 3 mM BME and centrifuged for 18 h at 110 000 × g in a Ti 50.2 rotor (Beckman Coulter) at 4 °C. The ribosome pellets were washed and dissolved by gentle stirring in the same buffer without sucrose and subjected to a second sucrose cushion centrifugation as above. Ribosome pellets were resuspended in buffer X (20 mM Tris–HCl pH 7.5, 60 mM NH_4_Cl, 5 mM magnesium acetate, 0.25 mM EDTA, 3 mM BME), and the 70S ribosomes were isolated by zonal centrifugation in a Ti 15 rotor (Beckman Coulter) for 15 h at 75 000 × g on a sucrose gradient from 10 to 40% in buffer X. The 70S peak was collected, and the ribosomes were pelleted by ultracentrifugation in a Ti 50.2 rotor (Beckman Coulter) for 19 h at 180 000 × g, resuspended in HEPES polymix buffer (5 mM HEPES pH 7.5, 5 mM NH_4_Cl, 5 mM Mg(OAc)_2_, 100 mM KCl, 0.5 mM CaCl_2_, 8 mM putrescine, 1 mM spermidine, and 1 mM dithioerythritol)^40^, shock-frozen in liquid nitrogen and stored at − 80 °C.

### Complex preparation

mRNA Z4AUGGCA (5′-GGCAAGGAGGUAAAAAUGGCAAAA-3′) was produced by chemical synthesis (Dharmacon) and *E. coli* tRNA^fMet^ was overexpressed and purified as published^41^. The FA complex was prepared by mixing 0.5 µM (final concentrations) 70S *S. aureus* ribosomes in HEPES polymix buffer (with 20 mM HEPES pH 7.5 and 5 mM BME as reducing agent) with 2 µM mRNA Z4AUGGCA and incubated for 10 min at 37 °C. Then, 2 µM *E. coli* tRNA^fMet^ was added and the mix was incubated for 10 min at 37 °C. Next, 5 µM *S. aureus* EF-G, 400 µM FA (Sigma-Aldrich, Merck, Darmstadt, Germany), and 1 mM GTP were added and incubated for 10 min at 37 °C, and kept on ice until plunge-freezing. The FA-CP complex was prepared as above, but with 5 µM *E. coli* tRNA^fMet^, and 400 µM FA-CP^29^ instead of FA.

### Cryo-EM grid preparation and data collection

#### FA complex

A QuantiFoil 300-mesh R 2/2 grid with 2 nm continuous carbon (QuantiFoil Micro Tools GmbH, Großlöbichau, Germany) was glow-discharged 30 s at 20 mA and 0.39 mBar using an EasiGlow (Ted Pella, Inc., Redding, CA, USA). 2.5 µl of FA complex was incubated on the grid for 60 s. Then 1 µl of FusB 25 µM in polymix buffer with 400 µM FA was mixed into the drop, blotted for 4 s, and plunge-frozen in a Vitrobot mark IV (Thermo Fisher Scientific) at 4 °C and 95% humidity. The grid was screened at the Uppsala Cryo-EM Facility, Sweden on a Glacios TEM operated at 200 kV equipped with a Falcon-III direct electron detector (Thermo Fisher Scientific). The final data was collected at SciLifeLab in Solna, Sweden on a Titan Krios G2 (Thermo Fisher Scientific) operated at 300 kV and equipped with a K3 BioQuantum direct electron detector (Gatan, Inc, AMETEK, Berwyn, PA, USA) and energy filter using 20 eV slit. The data was acquired at 105 000 × nominal magnification with a calibrated pixel size of 0.824 Å. A 50 µm C2 aperture was inserted and the TEM was operated in nanoprobe mode at spot size 6 and 670 nm beam size. The movies were collected in 30 frames over 1.3 s with a total dose of 30.83 e^-^/Å^2^ (16.1 e^−^/pixel/s) with a set defocus between − 0.5 and − 1.5 µm.

#### FA-CP complex

A Quantifoil 200-mesh R 2/1 grid with 2 nm continuous carbon was glow-discharged 15 s at 20 mA and 0.39 mBar using an EasiGlow. 3 µl of FA-CP complex was incubated on the grid for 30 s, then blotted for 4 s, and plunge-frozen in a Vitrobot mark IV at 4 °C and 95% humidity. The grid was screened at the Uppsala Cryo-EM Facility as described above and the final data was collected at SciLifeLab in Umeå, Sweden in a Titan Krios G2 operated at 300 kV and equipped with a Falcon-4i direct electron detector and Selectris energy filter (Thermo Fisher Scientific) using 10 eV slit. The data was acquired at 165 000 × nominal magnification with a calibrated pixel size of 0.728 Å. A 50 µm C2 aperture was inserted and the TEM was operated in nanoprobe mode at spot size 6 and 520 nm beam size. The EER formatted movies were collected in 684 raw frames with a total dose of 27.77 e^-^/Å^2^ (12.45 e^−^/pixel/s) over 2.23 s with a set defocus between − 0.7 and − 1.3 µm.

### Cryo-EM data processing

#### FA dataset

The dataset was processed with cryoSPARC v3.3.1^28^ following the workflow in Figure S6 resulting in a 2.49 Å reconstruction of the FA complex in the chimeric state.

#### FA-CP dataset

The dataset was processed with cryoSPARC v4.0.2 and v4.1.2 following the workflow in Figure S7 resulting in a 2.02 Å reconstruction of the FA-CP complex in chimeric state and a 2.44 Å reconstruction of the FA-CP complex in classical state. The CTFs were estimated using CTFFIND4^42^.

The FSC was calculated using cryoSPARC with a mask that was generated by low-pass filtering each map to 20 Å, thresholding to a level that covers the whole complex (0.1 for FA CHI complex, 0.06 for FA-CP CHI complex, and 0.07 for FA-CP POST complex), dilated 4 pixels and soft-padded 8 pixels. The final maps were post-processed using local filtering on cryoSPARC v4.2.1. Local resolution maps were calculated using cryoSPARC.

### Model building

Model building was started by individually rigid-body fitting all chains from the 3.1 Å *S. aureus* 70S ribosome structure (PDBID: 7NHM) in ChimeraX v1.5^43^, excluding the tRNA into the FA-CP CHI state unsharpened map. 16S RNA was fitted in three different fragments (1–921, 922–1396, and 1397–1552). EF-G was rigid-body fitted from the 1.9 Å x-ray crystal structure of *S. aureus* EF-G 2XEX in two different fragments (1–401 and 402–692). The E-site tRNA was rigid-body fitted from the 3.3 Å *E. coli* mid-translocation intermediate ribosome structure (PDBID: 7SSD). All chains were manually inspected and adjusted in Coot v0.9.8.5^44^, followed by real-space refinement in Phenix v1.20.1-4487-000^45^ against the local filtered map using secondary structure and Ramachandran restraints. This model was used as starting point for the FA CHI and the FA-CP POST structures and refined following the same procedure. For the FA-CP POST structure, the P-site tRNA was rigid- body fitted from the 2 Å *E. coli* ribosome structure (PDBID: 7K00). Water molecules were added to the FA-CP structures using Coot. Coordination waters for magnesium ions were manually corrected. Model validation was performed using MolProbity^46^ and problematic areas were inspected and corrected. The B-factors were refined against the unsharpened maps using Phenix. All ligand restraints were produced using the Grade web server^47^. Hidden surface area calculations were made using areaimol from CCP4^48,49^. The structure figures were rendered using ChimeraX. Figure S4C, D was generated using LigPlot + ^50^.

### Supplementary Information

Supplementary Information.

## Data Availability

The cryo-EM maps and models in this study have been deposited in the Electron Microscopy Data Bank under the accession codes EMD-17365 (2.5 Å FA complex with chimeric pe/E tRNA), EMD-17363 (2.4 Å FA-CP post-translocational state complex), and EMD-17364 (2 Å FA-CP complex with chimeric pe/E tRNA), and Protein Data Bank under the accession codes 8P2H (2.5 Å FA complex with chimeric pe/E tRNA), 8P2F (2.4 Å FA-CP post-translocational state complex), and 8P2G (2 Å FA-CP complex with chimeric pe/E tRNA). Python custom scripts used during analysis and refinement are available on Git Hub (https://github.com/adriangzlz97/pdb_python_tools).
