# Supplementary material for: Structures of the Staphylococcus aureus ribosome inhibited by fusidic acid and fusidic acid cyclopentane
Source: Sci Rep. 2024 Jun 20;14:14253. doi: 10.1038/s41598-024-64868-x (PMC11190147; doi:10.1038/s41598-024-64868-x)
Supplement: Supplementary file 1 — Supplementary Information. [file 41598_2024_64868_MOESM1_ESM.docx]

**Supplemental material for:**

**Structures of the *Staphylococcus aureus* ribosome inhibited by fusidic acid and fusidic acid cyclopentane**

Adrián González-López^1^, Daniel S. D. Larsson^1^, Brett Nelson Cain^2^, Ravi Kiran Koripella^1,3^, Suparna Sanyal^1^, Paul J. Hergenrother^2^, and Maria Selmer^1^*

1. Department of Cell and Molecular Biology, Uppsala University, BMC, P.O. Box 596, SE-75124 Uppsala, Sweden
2. ﻿Department of Chemistry, University of Illinois at Urbana−Champaign, Urbana, Illinois 61801, USA
3. Present address: Robert P. Apkarian Integrated Electron Microscopy Core, Emory University, Atlanta, GA 30322, USA

*To whom correspondence should be addressed: maria.selmer@icm.uu.se, tel. +46 18 4714177

**Content:**

**Supplemental Figure S1-S7**

**Supplemental Table S1-S2**

**
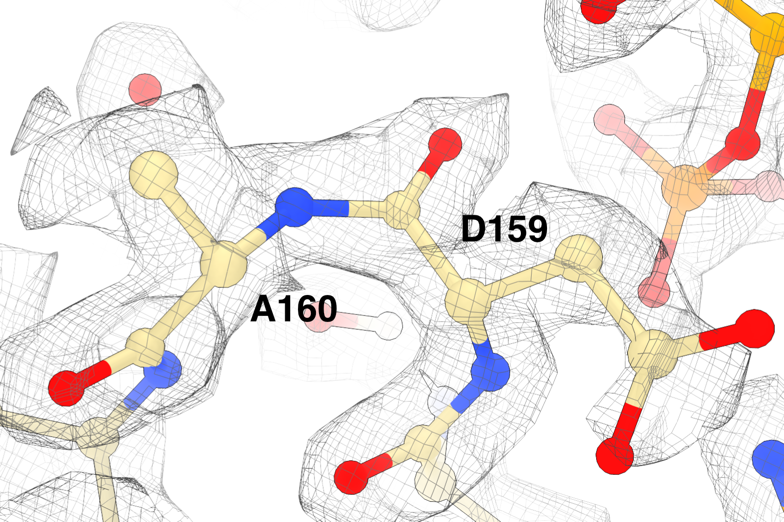
**

**Figure S1.** Cis peptide bond between residues D159 and A160 in *S. aureus* uL3.

**
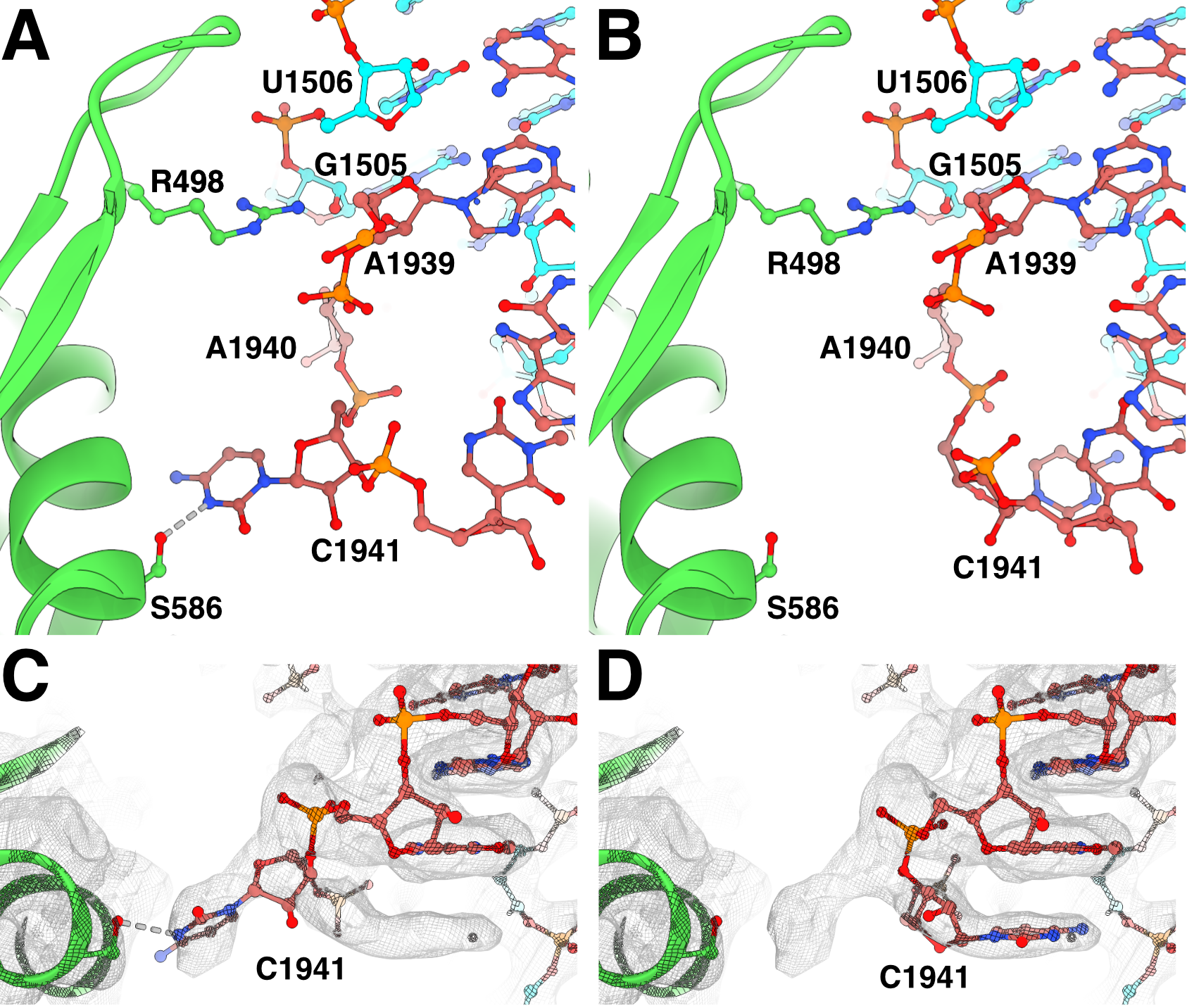
**

**Figure S2.** Interactions of EF-G (green) with 16S rRNA (cyan) and 23S rRNA (brown) at the intersubunit bridge B2a in the FA-CP CHI structure. C1941 shows two different conformations. **(A)** Conformation where C1941 interacts with EF-G. **(B)** Conformation where C1941 stacks with helix 69. **(C-D)** FA-CP CHI map around C1941 for both conformations.


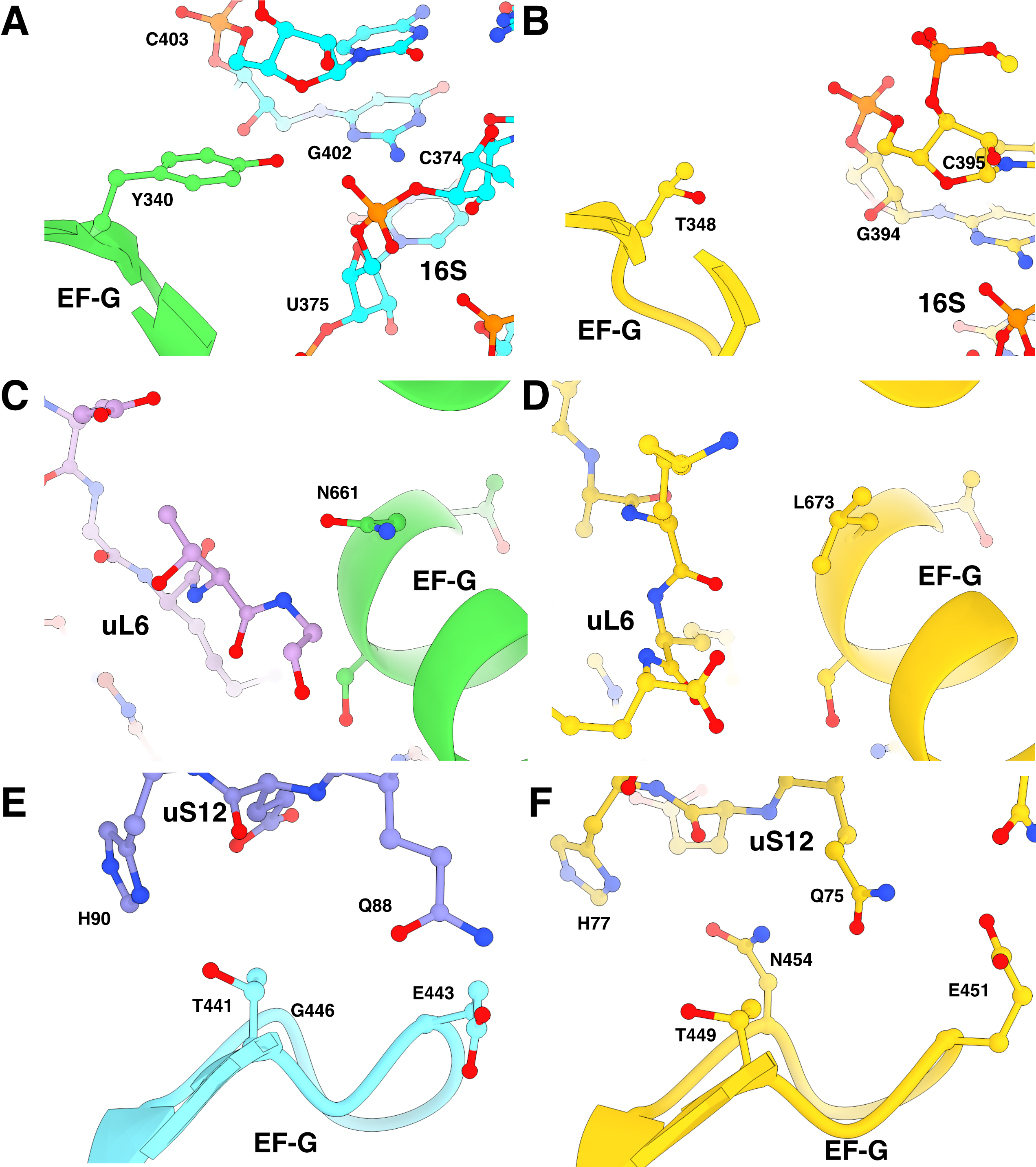


**Figure S3.** Differences in interactions of EF-G with the ribosome in *S. aureus* (this work, **A, C, E**) and *E. coli* (PDB ID 7N2C, **B, D, F**).


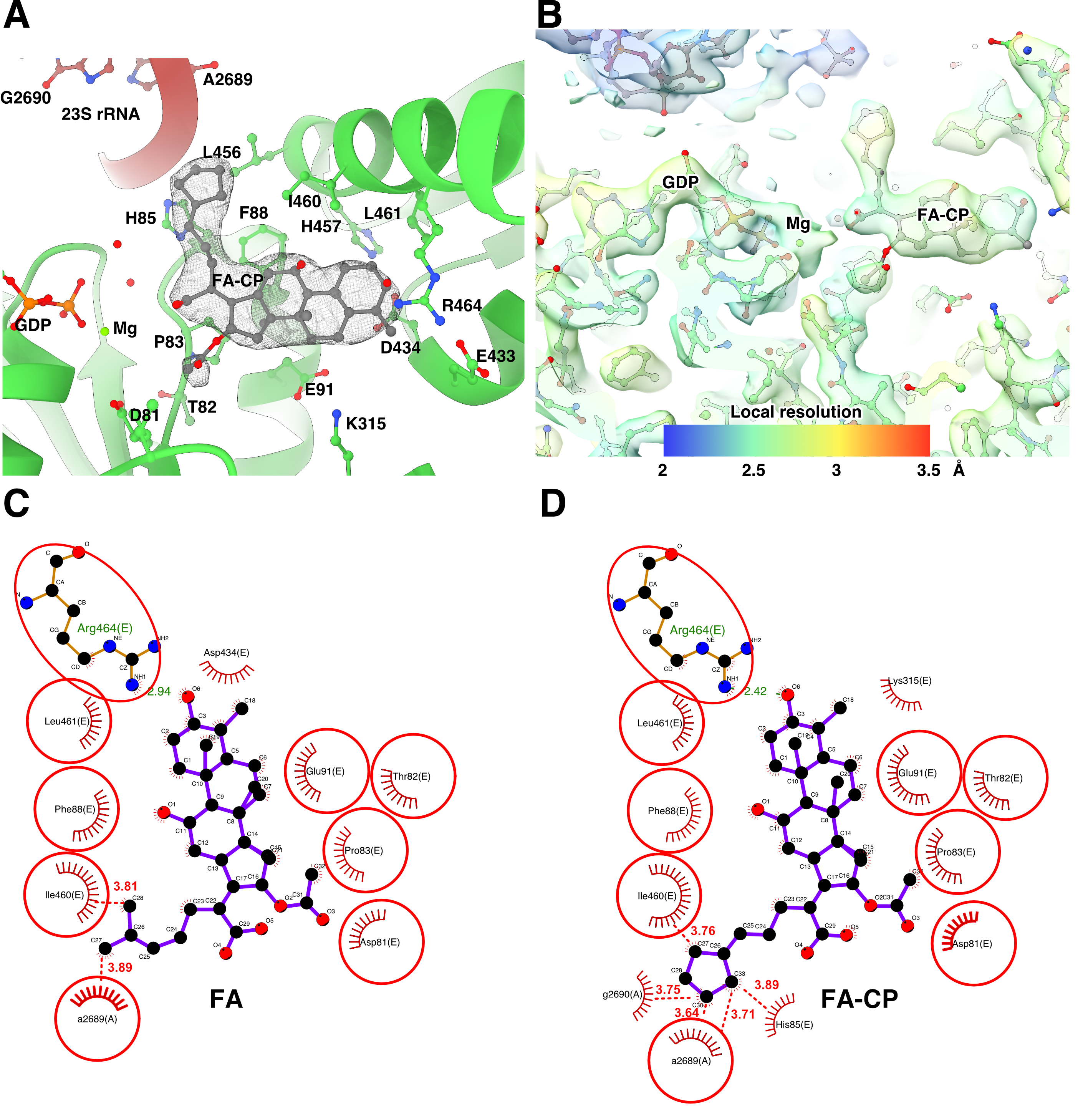


**Figure S4. Density of FA-CP and binding sites of FA-CP and FA (A)** Segmented local filtered map around FA-CP in POST state. Side chains within 4.5 Å of FA-CP are shown as sticks. **(B)** Local resolution map of the region around FA-CP and GDP in the FA-CP CHI state. The density clearly shows the octahedral coordination of the magnesium ion (coordination waters were not modeled). **(C-D)** Ligplot representation of the binding sites of FA and FA-CP. Residues within 3.9 Å of the ligands are shown in the figure. Residues from EF-G are indicated with (E) and residues from 23S rRNA with (A). Distances are indicated for the two methyl groups of FA and the cyclopentane group of FA-CP. Hydrogen bonding distances are indicated in green. Residues within 3.9 Å of both ligands are marked with a red circle.


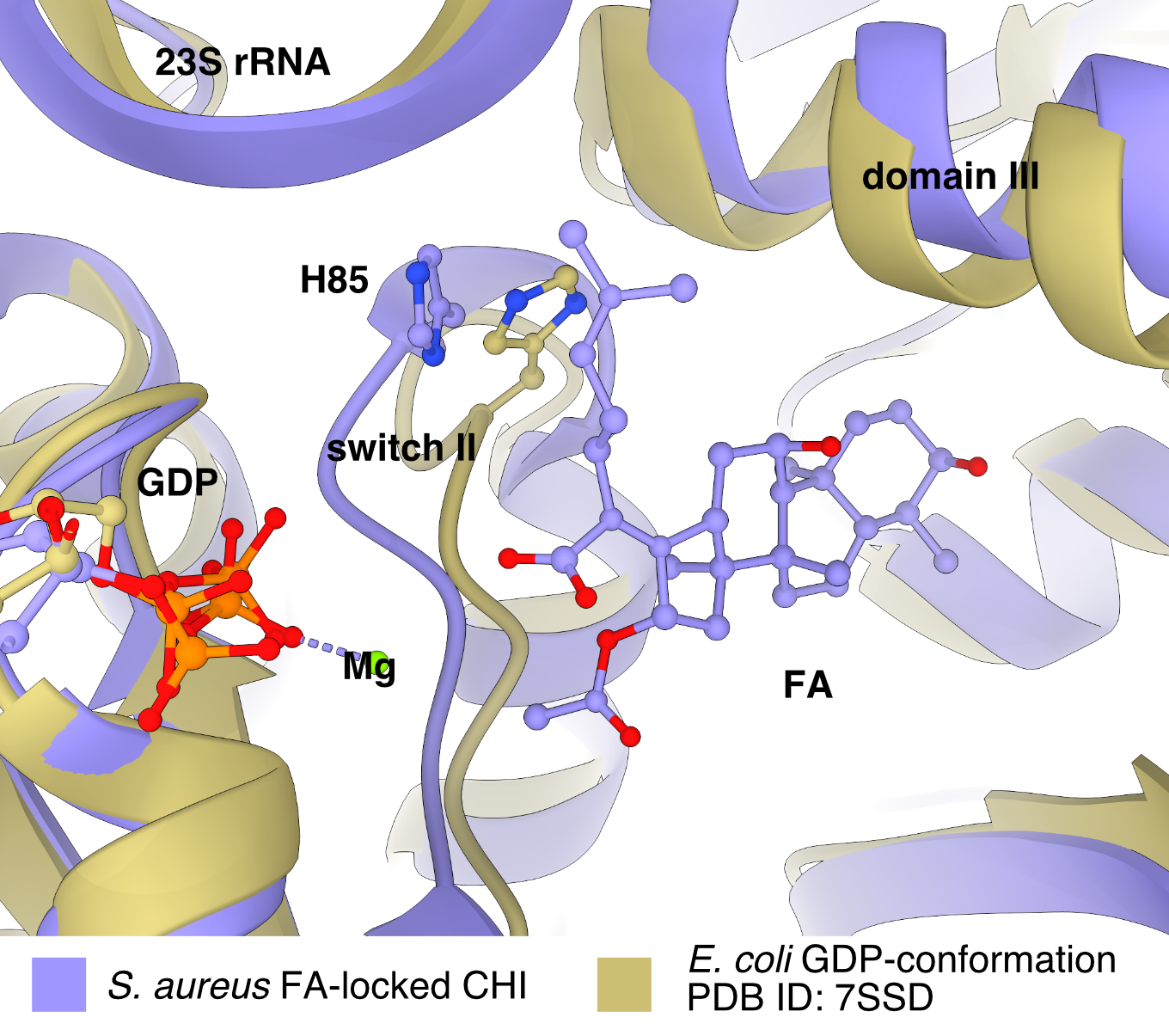


**Figure S5.** Comparison of switch II of EF-G between the GDP conformation of *E. coli* EF-G on the ribosome (PDB ID: 7SSD) and the FA-locked CHI structure from *S. aureus.*


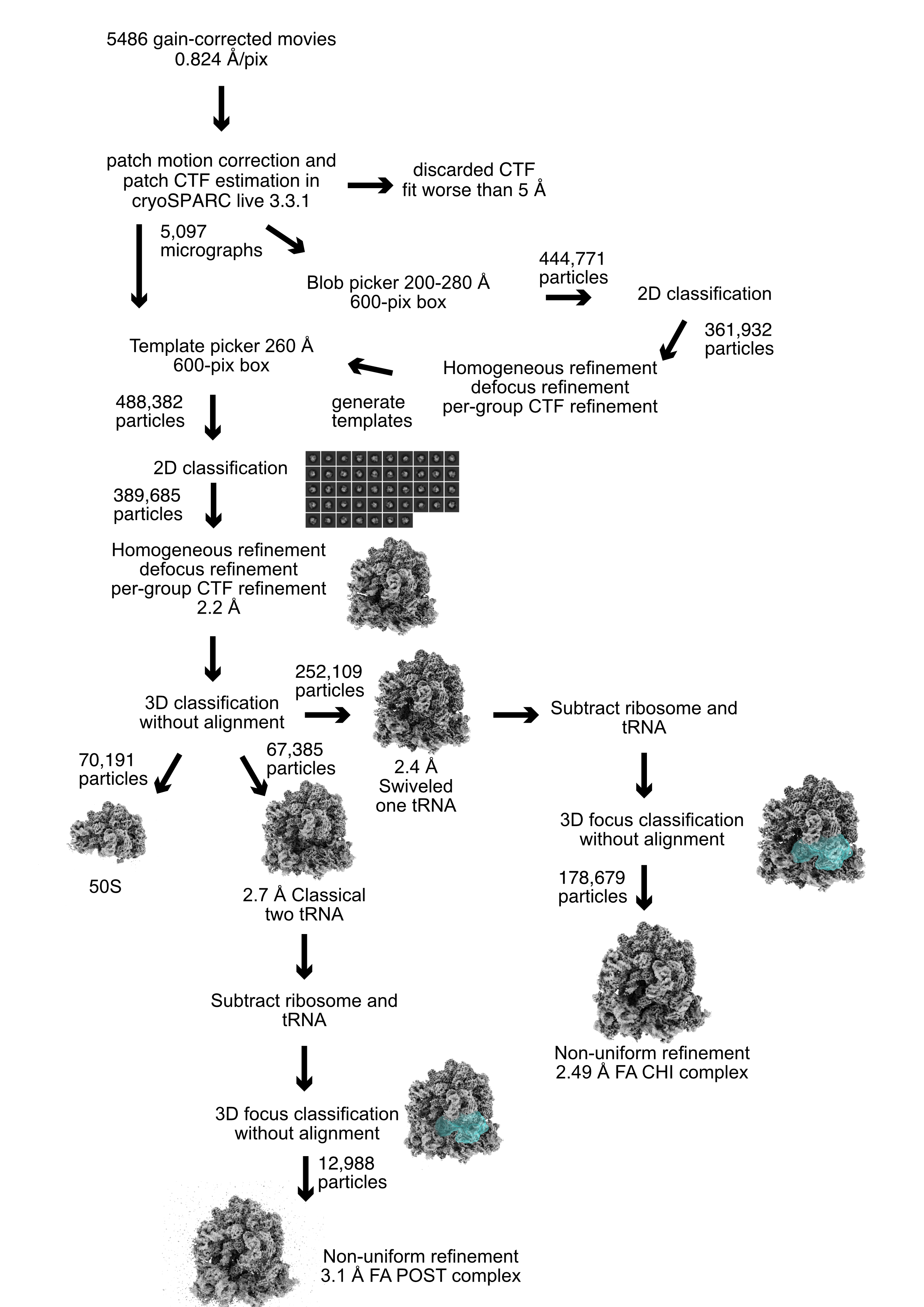


**Figure S6.** Cryo-EM workflow for the FA dataset. All steps were run with cryoSPARC v3.3.1+220118.


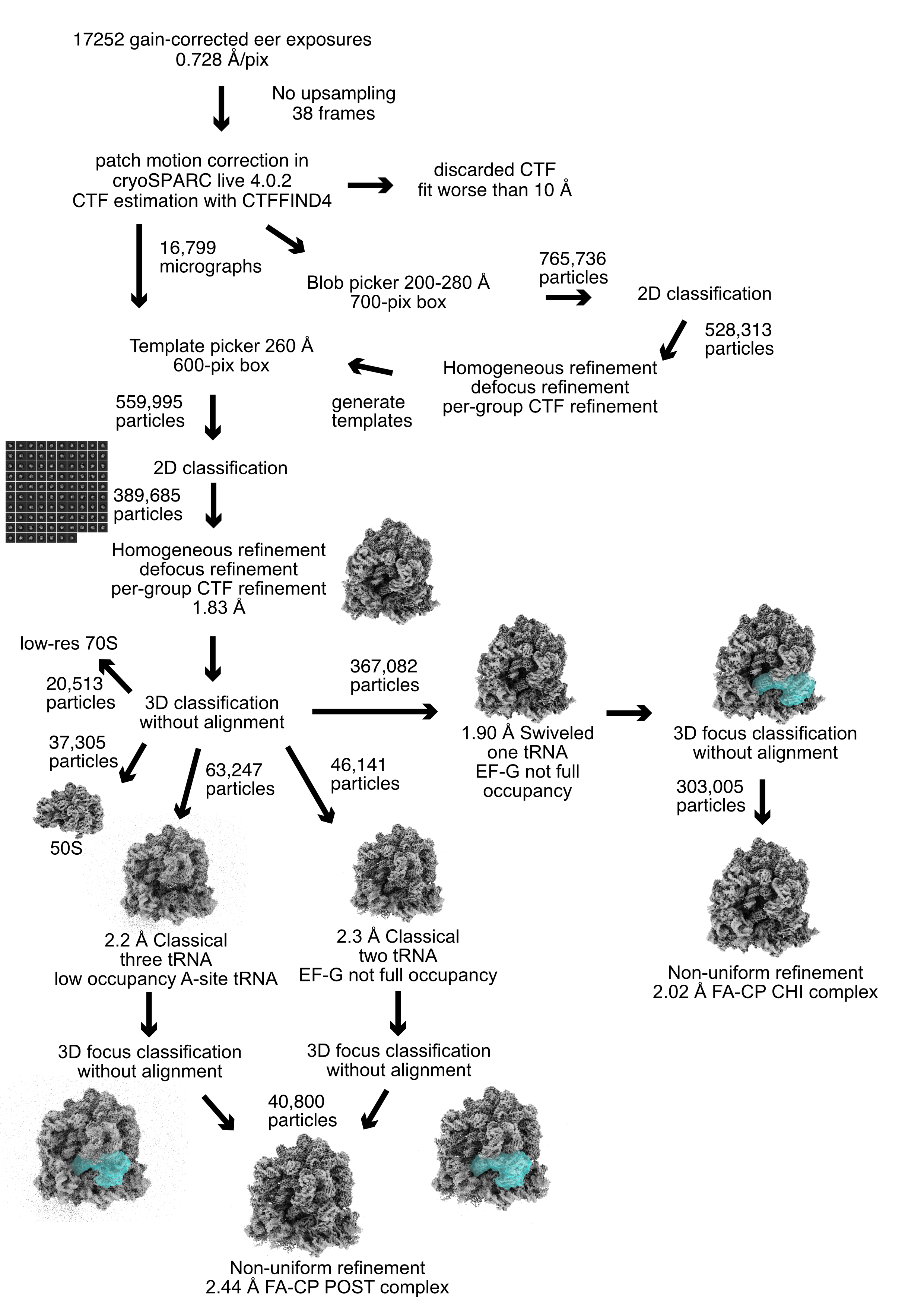


**Figure S7.** Cryo-EM workflow for the FA-CP dataset. All steps were run with cryoSPARC v4.0.2 or v4.1.2.

**Table S1.** Details of interactions of *S. aureus* EF-G with the ribosome.

| EF-G residue | Interacting residue | Type |
| --- | --- | --- |
| H18 | G2688 of 23S | Side chain to backbone phosphate |
| H85 | A2689 of 23S | Side chain to backbone phosphate |
| K324 | A367 of 16S | Backbone to backbone phosphate |
| Y340 | C403, G402 and U375 of 16S | Side chain to backbone phosphate and 2’OH |
| R349 | A404 of 16S | Side chain to backbone phosphate |
| R351 | U376 of 16S | Side chain to backbone phosphate |
| R354 | U376 of 16S | Side chain to base |
| D421 | R69 of uS12 | Side chain to sidechain |
| T441 | H90 of uS12 | Side chain to sidechain |
| E443 | Q88 of uS12 | Side chain to sidechain |
| R498 | A1940 of 23S and U1506 of 16S | Side chain to backbone phosphate |
| G530 | C1220 of 16S | Backbone to backbone phosphate |
| R535 | C1218 of 16S | Side chain to backbone phosphate |
| S586 | C1941 of 23S | Side chain to base |
| S660 | A2687 of 23S | Side chain to base |
| N661 | T176 of uL6 | Side chain to backbone |
| Q663 | A2687 of 23S | Side chain to phosphate |

**Table S2.** Cryo-EM data collection settings.

|  | FA | FA-CP |
| --- | --- | --- |
| Electron microscope | Titan Krios | Titan Krios |
| Detector | Gatan K3 | Falcon 4i |
| Calibrated Pixel size [Å/pixel] | 0.824 | 0.728 |
| Defocus range [µm] | -0.5 to -1.5 | -0.7 to -1.3 |
| Voltage [kV] | 300 | 300 |
| Energy filter slit width [eV] | 20 | 10 |
| Electron dose [e-/A2] | 30.83 | 27.77 |
| Number of micrographs | 5 486 | 17 252 |
